# The British Medical Association and Homœopathy

**Published:** 1881-11

**Authors:** 


### HOMŒOPATHIC PHYSICIAN.

A MONTHLY JOURNAL OF MEDICAL SCIENCE.

"If our school ever gives up the strict inductive method of Hahnemann, we are lost, and deserve to be mentioned only as a caricature, in the history of medicine."—CONSTANTINE HERING.

Vol. I. NOVEMBER, 1881. No. 11.

### THE BRITISH MEDICAL ASSOCIATION AND HOMŒOPATHY.

THE London *Lancet* has recently been directing its gigantic energies against homœopathy, and has demanded that the British Medical Association should enact stringent measures against all communion with such wretches as homœopathists. Homœopathy is dead, they say; yet it seems to require a great many obituaries and post-mortems to assure the timid allopathists of its demise. If it be dead, why not generously exclaim, *requiescat in pace*, and pass on to other and more live topics?

The disgraceful conduct of certain celebrated allopathists, when asked to render aid to a dying man—the Earl of Beaconsfield—has already wrought its legitimate results. The sarcastic denunciation of their conduct by the secular press has, at last, opened the long closed eyes of the allopathists, and they learn, doubtless to their disgust, that the laity not only tolerate, but admire and employ the despised homœopathists. Moreover, they observe that any high-handed bigotry on their part will but rebound to their own injury. Hence, in view of these observations, the British Medical Association hastens slowly to respond to the demand of the *Lancet*. If the speech of Dr. Bristowe, which we shall presently quote, is to be taken as an index of their views, we may say the members of that learned association quietly declined to put their heads into the hole dug for them by the *Lancet*. In so declining, the British Association evinces more wisdom than their confrères of the American Medical Association, by whose late action the now celebrated "Code" is evidently endangered. Nor will the case of President Garfield, its inception, treatment or termination, tend to ameliorate their perplexities.

Dr. Bristowe treats his subject—homœopathy—more rationally than is usual with allopathists. His address is very fair and candid—for an allopathist—and does him honor. Yet he, too, evades the real and only question at issue between the schools: *which cures best, quickest and easiest?* He evades this question. He pronounces our law, our theories, our doses, our founder, one and all, absurd. As to the absurdity of our law, he advances no argument; as to its inability to cure the sick, when properly applied, he gives no proof. All is mere assertion, mere chaff. The line of argument followed is much the same as that used by the late Sir John Forbes. Dr. Bristowe claims that homœopathy has been built up by persecution, and that tolerance will kill it. He says truly: "If false, as we believe it to be, its doom will be sealed when active antagonism and enforced isolation no longer raise it into fictitious importance." All hangs on that "IF." But suppose it be true? Will its toleration then contaminate the "regular?" We fear so. As to consultations between homœopathists and allopathists, we have yet to hear of a real homœopath desiring or *needing* one. It is only those of the Kidd stripe who can receive therapeutic aid from allopathy.

### BRITISH MEDICAL ASSOCIATION.

### ADDRESS IN MEDICINE.

JOHN SYER BRISTOWE, M. D.

The speaker began by alluding to the position of medicine among the sciences. He thought that those who criticised its work did not understand how vastly more difficult and complicated its study is than that of the exact sciences. As regards the future possibilities of medicine, he took no Utopian views. We shall never find specifics for every disease, or make mortal life immortal. But we can expect vast improvement over its present condition. He then took up the subject of

#### HOMŒOPATHY.

He sketched the life of Hahnemann, and then gave an interesting account of the Organon. "This Organon," he said, "is a remarkable work, very interesting also, and very entertaining; for it not only comprises the quintessence of his labors, but reveals the character of the man, as in a mirror, with all his strength and all his weakness, all his wisdom and all his folly.

"Hahnemann was a physician who had a supreme contempt for pathology, and, on the whole, for etiology. He inveighs over and over again against the absurdity of those who endeavor to discover, in morbid phenomena within the body, an explanation of the symptoms which persons who are ill present. He says: 'We may well conceive that every malady implies a change in the interior of the organism, but this change can only be surmised obscurely and fallaciously from the symptoms; it can never be recognized infallibly in its complete reality. The invisible changes wrought by the malady within the organism, and the changes perceptible to our senses (that is to say, the sum of the symptoms), together form a complete image of the malady; but that image is only visible in its entirety to the eye of the Creator. It is the totality of the symptoms which alone constitutes the part of it accessible to the doctor; but it is likewise in the totality of the symptoms, that we find everything that it is needful to know in order to cure.' To Hahnemann it is a matter of no moment whether ascites depends on cirrhosis of the liver, or tubercle of the peritoneum; whether an attack of constipation and colic arises from lead-poisoning or from a cancerous stricture; whether a paralytic seizure is the outcome of hysteria, or is due to some material lesion of the brain. In each case, to him, what is the condition of things within is an idle speculation; the symptoms of which the patient complains comprise all that the medical man need know; and to treat these according to the true laws of homœopathy is to cure the disease. But he goes further: for, not satisfied with stigmatizing all pathological investigations as mere pedantry and foolishness, he actually objects to all attempts on the part of systematic writers and practical physicians to distinguish and classify diseases.

"Hahnemann rejected therefore etiology as being of any use to the physician; he rejected pathology, or more especially, morbid anatomy and nosology.

"Hahnemann's views of the nature of disease were doubtless subservient to his views of the curative operation of drugs. And it is on his therapeutical views, if on anything, that his reputation must depend.

"Stated generally, his views are as follows: The innumerable diseases which afflict mankind, and which arise out of natural causes, consist, for the purposes of the physician, of groups of symptoms; the innumerable remedial agents which exist in nature, locked up in the animal and vegetable kingdoms, and in the inorganic world, are themselves the causes of a parallel series of artificial diseases, which again, for the purpose of the therapeutist, consists of groups of symptoms; in order to cure any natural disease that may come before us, it is necessary to administer that particular remedial agent which is capable of producing identical symptoms with it, and of course this must be given in a suitable dose, for, if in too minute a dose, it leaves a residuum of the original disease uncured; if in too large a dose, it cures the disease, but induces after-effects of its own; and, further, inasmuch as we are not yet acquainted with the specific virtues of all remedies, and inasmuch therefore, as for a large number of diseases the most suitable homœopathic remedy has not yet been discovered, we must in such a case select a remedy the effect of which approximates to the symptoms of the disease, by which means we shall cure a certain area, so to speak, of the primary disease, but we shall leave anew disease behind, compounded of the as yet uncured symptoms of the old disease, and the supernumerary symptoms due to the drug itself, which new disease must be treated *de novo* on homœopathic principles. How curious, how ingenious, how interesting the whole thing is! How excellent, if true! And has it not the simplicity of truth in it? The entire range of diseases, the entire range of therapeutics, converted into Chinese puzzles; the phenomena of diseases and the effect of drugs upon them treated as algebraical equations! It is impossible to conceive of any physician working daily by the bedside of patients, and in the dead-house, and seeing diseases as they are, framing such a system, except as a joke. It could only have been, as in fact it was, the serious work of a visionary who had thrown off the trammels of fact, and, allowing his imagination to run riot, mistook its fantastic figments for a revelation from Heaven.

"That Hahnemann believed in himself and in the absolute truth of all that he taught, is beyond dispute. He was a prophet, not only to his followers, but in his own eyes. All other systems of therapeutics but his were folly, and all who pursued them were fools. That he had learning, and ability, and the power of reasoning, is abundantly clear. He saw through the prevalent therapeutical absurdities and impostures of the day; he laughed to scorn the complicated and loathsome nostrums which, even at that time, disgraced the pharmacopœias; and he exposed with no little skill and success the emptiness and worthlessness of most of the therapeutical systems which then and theretofore had prevailed in the medical schools; and then he invented and proclaimed a system of his own at least as empty and as worthless as any that had gone before. In this, I suppose, there is nothing very strange; for it is only the broadest intellects (and his was an essentially narrow one) which are capable of treating the offspring of their own brains with the severe impartiality they manifest in other cases."

The speaker then discussed the various arguments and facts alleged to support the homœopathic doctrine. He referred to the meagre and unreliable statements of the Organon and the contradictions found there; the great and inevitable sources of error in the Hahnemann system of proving and of recording cases; the astonishing theory of infinitesimal doses.

He referred then to some of the changes that had been brought about in

MODERN HOMŒOPATHY.

One of these is the hypothesis which converts homœopathy into antipathy. It is to the effect that all medicines have opposite effects, according as they are given in large or in small doses, and that when, as the consequence of proving on the healthy person, a drug is found to excite the symptoms of a disease, it cures that disease by its opposition to it when given in small doses.

The speaker showed also that homœopathy had as yet furnished no genuine and reliable statistics as to its therapeutical value.

Dr. Bristowe concluded with a discussion of the relations between homœopathy and orthodox medicine.

"So far, gentlemen, I have discoursed only on homœopathy as a science and an art. I wish to add a few words on homœopathists as men, and as members of our common profession.

"That a very strong feeling of hostility should have arisen early between orthodox practitioners and homœopathists is not to be wondered at, when we consider, on the one hand, the arrogance and intolerance which Hahnemann displayed, at any rate in his writings, and on the other hand the contempt which experienced physicians felt and freely expressed for him and his whimsical doctrines. Nor is it to be wondered at that this variance should still be maintained; for homœopathy is still a protest against the best traditions of orthodox clinical medicine; and there is a natural tendency among us still to look upon homœopathic practitioners as knaves or fools. But surely this view is a wholly untenable one. That all

HOMŒOPATHISTS ARE HONEST MEN

is more than I would venture to assert; but that in large proportion they are honest is entirely beyond dispute. It is quite impossible that a large sect should have arisen, homœopathic schools and hospitals have been established, periodicals devoted to homœopathic medicine be maintained, and a whole literature in relation to it have been created, if it were all merely to support a conscious imposture. No, gentlemen, the whole history of the movement and its present position are amply sufficient to prove that those, at any rate, who take the intellectual lead in it are men who believe in the doctrines they profess, and in their mission; and who practice their profession with as much honesty of purpose and with as much confidence in their power to benefit their patients as we do. That all homœopathic practitioners are men of ability and education it would be absurd to maintain; but it is absolutely certain that many men of ability and learning are contained within their ranks. If you care to dive into homœopathic literature, you will find in it (however much you may differ from the views therein inculcated) plenty of literary ability; and I have perused many papers by homœopaths on philosophical and other subjects unconnected with homœopathy which prove their authors to be men of thought and culture, and from which I have derived pleasure and profit. Again, I will not pretend that even a considerable proportion of homœopaths are deeply versed in the medical sciences; yet they have all been educated in orthodox schools of medicine, and have passed the examinations of recognized licensing boards; so that it must be allowed that they have acquired sufficient knowledge to qualify themselves for practice. And some among them possess high medical attainments.

"But it may be replied, if these men are honest and educated, and at the same time duly qualified practitioners in medicine, how can they believe, and how can they practice, such a palpable imposture as homœopathy? Well, gentlemen, it is very difficult to account for the beliefs and

VAGARIES OF THE HUMAN INTELLECT.

It is only occasionally that our convictions are the result of conscious reasoning. For the most part they arise in the mind, and take possession of it, we know not how or why; and our reasonings in regard to them (if we reason at all) are merely special pleadings prompted by the very convictions they seem to us to determine—in other words, they are not the foundations of our beliefs at all, but exhalations from them. It is not surprising, therefore, that, even on matters of supreme importance, irreconcilable differences of opinion prevail, aye, amongst men of high integrity and cultivated intellect. And if we desire to live broad and unselfish lives, we must be slow to condemn all those who entertain convictions which to us seem foolish or mischievous and logically untenable, or to refuse to co-operate with them.

"There are few, even of the best among us, who have not weak points in intellect or character. And it would be deplorable, indeed, if, for example, those of us who look on spiritualism as one of the grossest follies of the times in which we live, were to scout the distinguished chemists and the great writers who devoutly believe in it; or were to refuse to do homage to the conspicuous abilities and high character of a great judge, because, throwing off the judicial impartiality which befits a judge, and acting under the influence of prejudice, emotion and ignorance, he has made himself the leader of all the hysterical sentimentalism of the day in a crusade against experimental physiology in this land of Harvey and of Hunter! The remarks just made apply especially to beliefs in relation to those matters which are incapable of exact scientific proof, and in which the feelings are largely involved—pre-eminently, therefore, to religion, to politics, and to medicine.

"I ask you, gentlemen, to forbear with me, if I push my arguments to their logical conclusion, and venture now to express an opinion which is opposed to the opinion which many, perhaps most, of you entertain. I do not ask you to agree with it; still less do I ask you to adopt it. But I ask you to consider it; and I am content to believe that, if it be just, it will ultimately prevail. It is that, where homœopathists are honest, and well-informed, and legally qualified practitioners of medicine, they should be dealt with as if they were honest and well-informed and qualified.

"I shall not discuss the question whether we can, with propriety or with benefit to our patients, meet

HOMŒOPATHS IN CONSULTATION.

I could, however, I think, adduce strong reasons in favor of the morality of acting thus, and for the belief that good to the patient would generally ensue under such circumstances. I shall not consider at length whether the dignity of the profession would be compromised by habitual dealing with homœopathists. But I may observe that it is more conducive to the maintenance of true dignity to treat with respect and consideration, and as if they were honest, those whose opinions differ from ours, than to make broad our phylacteries and enlarge the borders of our garments, and wrap ourselves up, in regard to them, in Pharisaic pride. I appeal, gentlemen, in support of my contention to other considerations. It has been held, that to break down the barriers that at present separate us from homœopathists, would be to allow the poison of quackery to leaven the mass of orthodox medicine. But who, that has any trust in his profession, any scientific instinct, any faith in the ultimate triumph of truth, can entertain any such fear? All the best physicians of old times, all the greatest names in medicine of the present day, are with us, all science is on our side; and we know that, as a body, we are honest seekers after truth. What have we to fear from homœopathy? Bigots are made martyrs by persecution; false sects acquire form and momentum and importance mainly through the opposition they provoke. When persecution ceases, would-be martyrs sink into insignificance; in the absence of the stimulus of active opposition, sects tend to undergo disintegration and to disappear. The rise and spread of homœopathy have been largely due to the strong antagonism it has evoked from the schools of orthodox medicine, and to the isolation which has thus been imposed on its disciples. If false, as we believe it to be, its doom will be sealed when active antagonism and enforced isolation no longer raise it into fictitious importance. At any rate, breadth of view and liberality of conduct are the fitting characteristics of men of science."

In the subsequent discussion, Dr. Long Fox criticised Dr. Bristowe's last remarks and said: If a homœopathic practitioner, alleging that the same remedies were used, the difference being only in name, asked an honorable member of a most honorable profession to associate with him in the treatment of a case, it appeared to him to be like asking the Archbishop of Canterbury to associate with the high priest of the lowest fetish in Central Africa. Why were the adherents—he would not say the victims—of homœopathy to be found among men eminent in piety, sanctity and benevolence? He believed it was really because they thought that God acted habitually miraculously. But as a reverend profession (as Bishop McDougall had called them), they ought to refuse to countenance so unphilosophical a view of the great First Cause. It was surely a much grander view of the Almighty to believe that He always acted by the grand laws that He had Himself laid down. He hoped that Dr. Bristowe would not suppose that he disagreed with anything he had said. He ventured only to differ in regard to the remarks in the latter portion of the address.

Dr. Bristowe, in reply, said he knew he had expressed some views not shared by the majority of the members, and he was not surprised at the observations his friend, Dr. Fox.—*Medical Record.*

Also note the remarks of Mr. Barrow, the President:

"No one can, I think, deny the homœopath stands upon very peculiar ground. He practices a system of medicine (although I have no belief in it), nevertheless it is a *system;* and, if carried on in its purity, as laid down by the founder of the system, and as long as the homœopath adheres strictly thereto, I fail to see how he can be called a quack, or why he should be tabooed by the profession; as it were, cut off from a position among medical men, forbidden to gather with them, and prevented from discussing publicly his system, and hearing the contrary from those practicing legitimate medicine. The benefit would be mutual, and these discussions would be of benefit to the public, and an additional proof to them that their weal was uppermost in our minds."

Careful readers will note that Mr. Barrow (an allopathist) considers homœopathy a system, while Dr. Hughes (a self-styled homœopath) calls it a *method!* The allopathist evidently knows more of true homœopathy than, *Richard Hughes*, the *pharmaco-dynamist!*

